# The influence of “industry policy” and “financial institution” configuration effect on innovation performance of China’s biomedical industry-based on necessary condition analysis and qualitative comparative analysis

**DOI:** 10.3389/fmed.2023.1297495

**Published:** 2024-01-08

**Authors:** Yaqiong Zhang

**Affiliations:** China University of Political Science and Law, Changping District, China

**Keywords:** biomedical industry, government policies, financial institutions, innovation performance, NCA and QCA

## Abstract

Biomedical industry is a strategic emerging industry in China, especially the outbreak of the Covid pandemic. The biomedical industry is characterized by high risk, high investment, high technology and long cycle, and each stage contains risks and challenges. How to optimize the policy environment and financial environment, explore the unique “policy” and “finance” model for the development of the biomedical industry, and improve the innovation performance has become an important issue. This paper analyzes the relationship among industry policy, financial institution and innovation performance in the biomedical industry from the configuration perspective, combining necessary condition analysis (NCA) and qualitative comparative analysis (QCA) research methods, using the A-share listed enterprises in Shanghai and Shenzhen in the biomedical industry from 2012 to 2020 as the research objects. It is found that (1) individual policy preference or financial institution dimension cannot constitute a necessary condition for generating high innovation performance of biomedical company, but increasing tax incentive and raising the proportion of equity-based financing method play a significant role in generating high innovation performance; (2) four “political” and “financial “synergistic grouping paths can generate high innovation performance, including tax incentives, financial institutions’ professional level and institutional background synergistic drive type; government subsidy, financing method and financial institutions’ professional level synergistic drive type; tax incentive and financing method synergistic drive type; tax incentive and institutional background drive type. Different synergistic grouping paths represent various ways to achieve high innovation performance of biomedical enterprise. In addition, the results show that the two “political” and “financial” groupings lead to low-to-medium innovation performance, which indicates that industry policy plays a very important role in the innovation performance and that the government’s support for emerging industries through policy is a significant force for the innovation development. This paper introduces the “political” and “financial” aspects to investigate the configuration effect of industry policy and financial institutions on the innovation performance of biomedical enterprise. The findings have important theoretical and practical implications for revealing the synergistic path of high innovation performance in the Chinese biomedical industry.

## 1 Introduction

As one of the strategic emerging industries in China’s 13th Five-Year Plan, the biomedical industry is regarded as the most promising emerging high-tech industry in the 21st century (1). With the increase of China’s total population and the aging of society, especially the recent trade friction and the outbreak of the Covid pandemic, medical needs have increased rapidly, and the national attention to the development of the biomedical field has been raised to an unprecedented height. Technological innovation is a global trend in the development of the biomedical industry, and the lack of innovation remains a key factor hindering the performance and development of Chinese biomedical enterprises. At present, more than 95% of China’s nearly 170,000 drug approval are generic drugs, and the problem of insufficient innovation is receiving attention from the government and investment institutions (1). The biomedical industry is characterized by high risk, high investment, high technology and a long cycle (2). How to improve the innovation performance, promote the transformation of results, and finally achieve technological breakthroughs and solve the problem of technology blockade is urgent for Chinese biomedical industry. Each stage of the biomedical industry contains high risks and challenges, in order to realize the breakthrough development of the biomedical industry rapidly, concerted efforts from all sides “government” “industry” “university” “research” and “financial institute” are needed (3).

From the existing literature, there is a wealth of research on innovation performance in the biomedical industry (4). Most of them are focus on the effects of “industry-university-research” synergy and “government-industry” synergy on innovation outcomes and performance (5). However, few studies have focused on the effects of the synergistic grouping of government policy and financial institutions on the innovation and performance of the biomedical industry. Why is the “policy-financial combination” seldom mentioned by scholars, and does the formulation of industry policies to support the industry affect the preference of financial institutions to invest in company? From the opposite point of view, it is worth exploring whether the presence of financial institutions can sustainably, deeply, and complementarily help biomedical company to maintain and improve the innovation power in the case of insufficient industry policy support and persistence.

From existing studies, industry policies closely related to the biomedical industry include government subsidy and tax incentive (6, 7). However, the implementation of industrial policy by the government has both positive and negative impacts on the innovation and performance of biomedical industry: on the one hand, the support of industrial policy will guarantee the development of biomedical enterprises, but it is difficult to motivate enterprises to continuously invest in R&D due to the limitation of policy support, which leads to a delay in the development of technological innovation; industry policy can also cause resource mismatch, resulting in a significant reduction in resource allocation efficiency (8). In the process of enjoying benefits of industry policy, if biomedical company can introduce suitable investment institutions and financial support in time, the problem of insufficient R&D funds in the process of expansion and development can be solved, and continuously maintain R&D investment, which will greatly promote and guarantee the accumulation of power and long-term efficient innovation development.

Similar to the policy impact, from the level of financial institution participation and capital investment, the choice of financing method, the background of financial institution, and the professional level of financial institutions have different effects on the innovation performance of biomedical industry. Compared with the perfect financing system in developed countries, the Chinese biomedical industry is affected by the constraints of the financing system such as the lack of innovation in financing methods, intellectual property rights and patented technologies. In addition, the financing channels are relatively limited and the financing efficiency is relatively low (7). In view of the above financing problems, if the Chinese government can efficiently and precisely support the biomedical industry in terms of policies, it will become a strong shot for financial institutions to make investment decisions, which will not only improve the problem of imperfect financing system in China through the national level but also help the biomedical industry to solve the problem of insufficient capital.

To achieve a virtuous cycle and healthy development of the capital chain and innovation of biomedical industry, the government and financial institutions need to make concerted development and efforts. The government needs to play the role of bridge and link between financial institutions and companies by optimizing the business environment and strengthening services so as to realize the effective docking between “financial” and “company.” On the other hand, financial institutions need to actively respond to the government’s policy focus on supporting the industry, providing a variety of services for biomedical companies, taking the initiative to dock the project, and taking practical action to support government policies. So, how to optimize the policy environment and financing environment, explore the unique “policy” and “financial” grouping mode suitable for the development of the biomedical industry, improve the innovation efficiency and further improve the performance has become an important issue for China’s biomedical industry. Although existing studies have explored various aspects of innovation in the biomedical industry, most of them have focused on the industry-university-research model and the influence of a single dimension on the innovation effect. In addition, most of the existing relevant studies focus on qualitative analysis, case studies and empirical studies, and few studies take a group perspective to analyze the configuration effect of policies and financial institutions on the innovation effect of biomedical enterprises, which cannot reveal the pervasive problems of China’s biomedical industry in a more comprehensive way. Therefore, this paper aims to explore and study the existing specific dimensions of “policy” and “financial,” and analyze the necessary and sufficient causal relationships through the combination of Necessary Condition Analysis (NCA) and Fuzzy Qualitative Comparative Analysis (fsQCA). It is important to explore the degree of influence of individual elements of “policy” and “financial” on the innovation performance and the configuration effect of multi-factor grouping so as to explore how to improve the innovation of biomedical industry in China in multiple paths and principles of action.

## 2 Literature review and model construction

### 2.1 Literature review

With the development of modern biological and pharmaceutical technology, the pharmaceutical industry supported by biotechnology has become one of the most promising industries and is the focus of R&D in various countries (7). From a domestic perspective, the stable macroeconomic environment, gradually favorable medical reform policies, and the people’s growing demand for health together promote the biomedical industry continually growth, and the growth rate is expected to remain at about 15% (4). At present, a large number of provinces and cities in China have taken biomedicine as a pillar industry, and are planning the layout in terms of R&D, development direction, industrial environment and policy incentives (9).

#### 2.1.1 Research on biomedical industry policies and innovation performance

The uniqueness of the biomedical industry determines that its innovation and development cannot be achieved without the support and promotion of government policies. However, the policies related to the biomedical industry in China are lag behind those in Europe and America (1). After the 13th Five-Year Plan, China has elevated the development of the biomedical industry to a whole new level. With the increasing importance of the biomedical industry, Chinese government has made diversified adjustments and implementation of policy support (6). In addition, countries around the world have attached great importance to the development of the biomedical industry, in which governments and policies play a crucial role (10, 11).

The relationship between industrial policy and innovation in biomedical and other high-tech industry has been well explored in existing studies in China and other countries. From the overall perspective of policies related to biomedical industry, it is pointed out that there is a causal relationship between innovation and industrial policies and that industrial policies can effectively guide the development and investment trends of companies, thus influencing their investment in R&D and promoting their innovation performance (10–12).

In China, some scholars argue that industrial policy can guide the development direction of the market and regulate the shortcomings of market mechanisms in technological industries (12). Especially in the process of financing, industrial policy can effectively help company to connect with investment institutions and improve the efficiency of investment, financing, M&A and production innovation (13). In addition, the Chinese government’s implementation of industrial policy can promote the rapid development of key national support areas, improve technological advantage in the international arena, and accelerate the solution of the “neck” problem, thus further promoting the rapid take-off of China’s economic development (12). However, on the other hand, scholars point out that because the government’s industrial policy has a certain “directionality,” it will directly interfere with the development of the market to varying degrees, resulting in the government’s will to affect the free law of market competition (1). Furthermore, the central government’s industrial policy will directly affect the local government’s adjustment of local industrial development planning, leading to a large area of government resources, financial institutions, and entrepreneurial enterprises in a certain field, resulting in a mismatch between advantageous local resources and key industrial development planning areas, bringing a negative impact on industrial and economic development (14). In addition, due to the existence of the industrial policy support stage, in the primary stage of enterprise’s innovation and start-up, it can indeed bring advantages and effectively motivate enterprises to invest in R&D and innovation. However, as the scale of company grows, it is difficult to guarantee continuous support from the industrial policy. This phenomenon can lead to a lack of funds, resulting in a shortage of continuous R&D investment and a significant decrease in the efficiency and results of innovation (14).

On the other hand, many countries around the world have also provided a great deal of support for the biopharmaceutical industry in terms of policies (15, 16). For example, US biomedical policies are categorized into national and state-level policies. At the national level, the federal government mainly provides research funds, establishes and improves laws and regulations related to intellectual property rights and industry-research cooperation, approves biomedical products, and supports and encourages venture capitalists to invest in biomedical industry. Each state provides funds for the development of the biomedical industry according to its own conditions and needs, promotes regional industrial cooperation, regulates taxes, and provides human resources for the development of the industry (17). The EU and EU countries have relatively conservative policies and regulations in the biomedical industry. In the EU and its member states, the development of biomedical technology has aroused widespread public concern and become a high-profile public issue. In turn, public attitudes, especially the general European distrust and skepticism toward biotechnology, have largely influenced the policy preferences of the EU and its member states. In the EU, biomedical science and technology is first and foremost understood as a safety and ethical issue, while economic development considerations are relatively downplayed. Accordingly, at the EU level, policies and regulations related to biomedical technology and industrial development are mainly focused on regulating and restricting the application of the technology. Most of the support policies in this field are formulated by individual member states according to their own situation, and countries generally play a relatively large role in promoting cooperation between industry and research (18). The development of Japan’s biomedical industry started later than that of Europe and the United States, and the government began to emphasize the development of the industry only after World War II, but its achievements are obvious to all. According to Trend Force’s study on the size of the global pharmaceutical market in 2018, Japan is the world’s third-largest pharmaceutical market, after the United States and China. Behind the take-off of the Japanese biomedical industry, the guiding policy of the Japanese government has played a very important role, and its government-industry-academia cooperation model is now very mature. The evolution of the whole policy can be summarized as “introduction-improvement-imitation-absorption-independent innovation” (19). In the above-mentioned studies on industrial policies, scholars have mainly discussed and studied the impact of government subsidy, tax incentive, and low-interest loans on the biomedical industry, especially in China (6, 8, 15, 18, 19).

First, in terms of government subsidies, a large number of scholars focus on the impact of government subsidies on R&D investment and innovation of biomedical enterprises (20). Chinese government provides direct resource support to enterprises by means of subsidies, which include R&D funding, research talents, industrial parks, and technology exchange (21). Using an empirical study, Huang Qi et al. analyzed the impact of government subsidies on firms’ innovation capacity and efficiency and found a “U” shaped relationship between the two (4). Similarly, a study by Liu et al. focused on firms’ innovation inputs and showed that different levels of government subsidies could have a facilitating or inhibiting effect on technological innovation inputs (22). As the Chinese government pays more attention to the biomedical industry, how to adjust and support it through policies has become a common concern for enterprises and academia (20). To this end, governments at all levels have been increasing their support for biomedical innovation, with direct subsidies being the main and most direct way. Related studies have found that credit mechanisms in strategic emerging industry policies have a positive impact on firms’ innovation performance, yet the impact of government subsidies is not significant (23). In addition, Kang and Park found that government subsidies for the biomedical industry stimulate R&D innovation within firms and cooperation between upstream and downstream of the industry. Secondly, government subsidies are policy-oriented and help enterprises to obtain financing and resource support, thus reducing their gown R&D investment, which helps to reduce their own risk aversion and stimulate their innovation motivation (24). Dimos and Leyden et al. show that government subsidies support firms’ R&D investment to reduce their own cost and increase their profit and expectation of R&D investment. This positively promotes the importance of R&D and innovation (25, 26). In addition, government subsidies indicate the development direction of key industries to the market in the most direct way, and enterprises will use the policy guidance to comprehensively assess the direction of project development and innovation, seize the opportunities brought by the favorable policy, and carry out targeted R&D and innovation activities (12).

In addition to government subsidies, tax incentives are also a major policy measure to stimulate R&D and innovation investment of enterprises (27). Existing domestic and international studies show that tax incentives have an incentive effect on innovation. Many scholars point out that tax incentives can promote R&D and innovation to a greater extent than government subsidies (28). In terms of the choice of tax incentives, direct tax incentives can help enterprises reduce their total costs and thus motivate them to invest more in innovation and R&D; while the effect of indirect tax incentives is reflected in the impact on the unit cost of enterprises, both of which will promote innovation and R&D (13). Reviewing the research on taxation and corporate innovation in China, similar to the research results of foreign scholars, Wu Jinming’s empirical study shows that tax incentives have a significant incentive effect on R&D and innovation of enterprises and are higher than government subsidies, among which the promotion effect of income tax incentives is more prominent, especially in high-tech industries (27). Qu Wan and Feng Haihong use tax incentives as the antecedent variable to investigate whether they have a positive effect on firms’ R&D investment (28). A review of the literature reveals that the existing tax-related studies generally show that the amount of negative tax is negatively related to the performance of firms while tax incentives are positively related to the R&D effort and innovation performance and further promote the performance (29, 30).

#### 2.1.2 Research on investment, financing and innovation performance of the biomedical industry

R&D innovation in the biomedical industry usually requires a long period of time, and companies need to invest a lot of human and material resources and capital, and the return period is also relatively long, while the free capital of enterprises cannot support independently, so they need a lot of external funding support (31). With the results of R&D innovation not yet clear, it is often difficult for investors to assess the degree of risk, return and future value of the project, resulting in the difficulty of financing biomedical enterprises (9, 32). Obtaining funds through financial investment institutions is one of the preferred financing methods for enterprises. Financial institutions have a high risk-taking ability and fault tolerance rate, and professional industry researchers, especially for emerging technology industries, have a greater investment preference and pay attention to the innovation ability of enterprises, which is an important support force to promote the development of the biomedical industry (31, 32). Under this premise, how to find a financing model suitable for the development of the biomedical industry becomes an essential prerequisite for improving the R&D and innovation results of the industry. At present, scholars have a certain basis for research on the biomedical industry, but it mostly focuses on industrial policy, R&D innovation and industrial cluster development, etc. There are relatively few studies on financing issues, especially the impact of different financing modes on enterprise’s innovation development is scarcer.

In the existing studies, scholars have empirically explored different financing preferences in the biomedical industry. The results show that the financing methods of listed biomedical enterprises are mainly equity-based and debt-based, and equity financing has become the main financing method for biomedical enterprises (3, 33). In terms of bond financing, enterprises prefer short-term debt-based financing (13, 33). There are advantages and disadvantages to both types of financing: equity-based financing will dilute the equity of the company to varying degrees, while bond-based financing does not require the surrender of equity but rather the payment of interest to obtain funds. Compared with bond-based financing, equity-based financing not only solves the problem of shortage of funds but also provides more resources and support from investors, such as guidance from professionals and endorsement from the reputation of investment institutions (9, 33). The presence of quality investment institutions can laterally reflect their confidence in the future development of the enterprise and potentially influence and motivate the innovation and development direction of the enterprises. Accordingly, equity-based financing also has disadvantages, and scholars argue that the presence of financial institutions, while providing positive improvements in corporate management, can reduce R&D and innovation activities due to the pursuit of stable growth and profits (33). Therefore, it is crucial for biomedical companies to have the flexibility to choose or combine both bond and equity financing to help them grow more rapidly.

In addition, the professional level and institutional background of financial institutions also affect the innovative development of biomedical enterprises (34, 35). Financial investment institutions have their own characteristics of selecting industry preferences and tend to invest in one or several industries. Financial institutions with experience in the biomedical industry have a deep understanding of the characteristics of this industry, focus on the technological innovation and future development potential of enterprises, evaluate enterprises by their R&D innovation intensity, and provide financial and technological support to selected companies to help them improve their innovation and revenue (34, 35). On the contrary, institutions that do not have experience in investing in the biomedical industry may place more emphasis on short-term interests, seek stable development of enterprises, accelerate the frequency of investment and financing, and obtain short-term income while ignoring R&D innovation of enterprises (36).

In terms of financial institution background, institutions with a government background are often able to quickly obtain a large amount of stable funding and policy support and are more sensitive to information on key industries supported by the state, so they can more efficiently select target enterprises and help them with sufficient resources of all kinds (37). In addition to considering investment returns, financial institutions with a government background also undertake the tasks of cultivating and developing emerging industries, pointing to key industries, promoting industrial upgrading and transformation, and promoting regional economic development. However, on the other hand, financial institutions with a government background are more rigid, complex and process-oriented in terms of system and process, so they may be influenced in the reverse direction in terms of investment strategy formulation and implementation, resulting in biomedical enterprises not receiving timely and efficient support, thus slowing down the momentum and efficiency of R&D and innovation behavior (12).

Therefore, how to optimize the development path of “political” and “financial” configuration effect of the biomedical industry and actively promoting the upgrading of the innovation process can effectively help China to shorten the gap and improve the innovation efficiency in the field of biomedicine, which is important for the development of the industry.

### 2.2 Model construction

**Figure 1:**
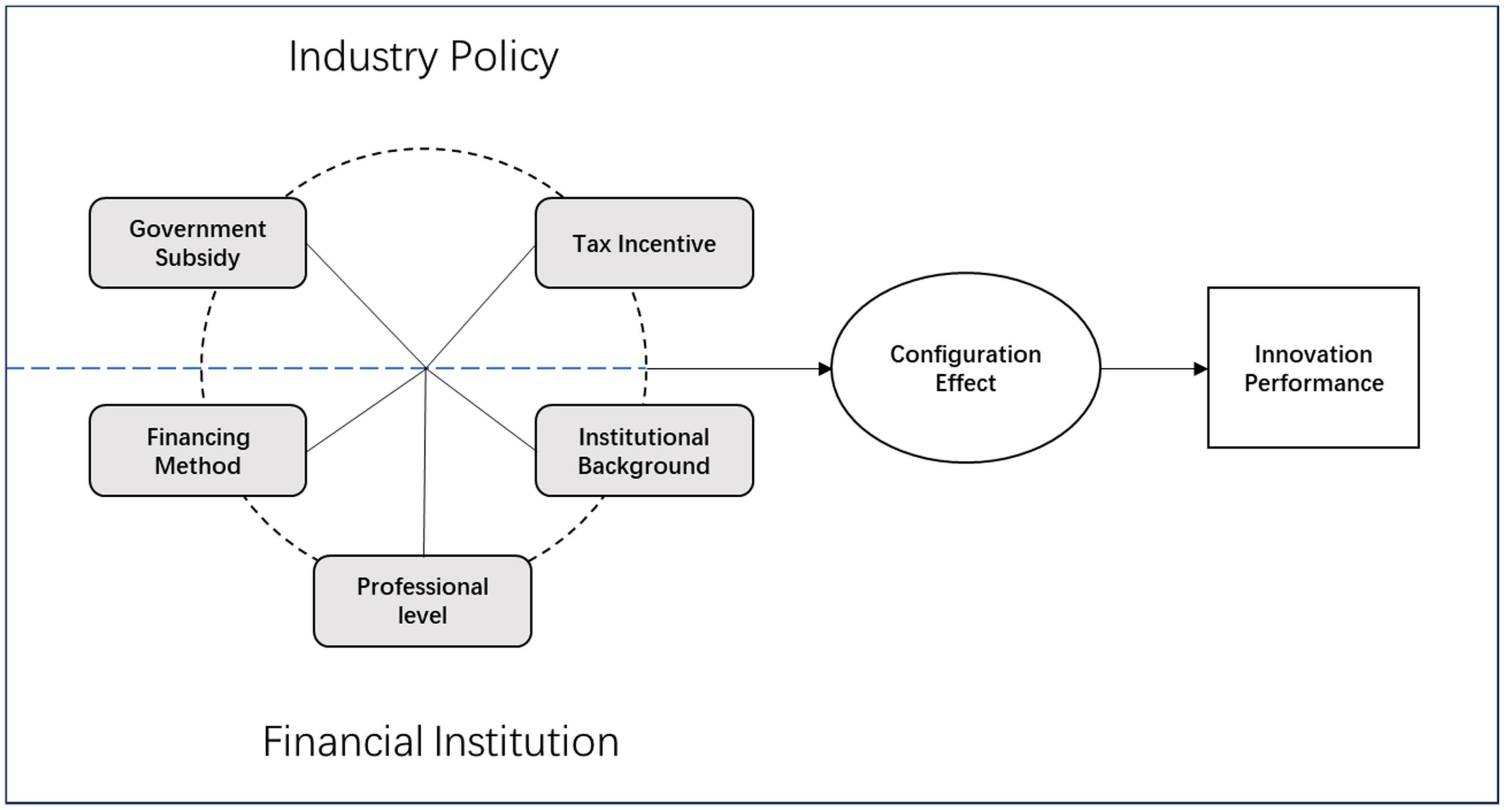
Study model.

The aim of this paper is to explore the configuration effect of dimensions influencing the innovation performance of biomedical enterprise. It is proposed to establish a total of five influencing factors in two dimensions, industry policy and financial institution, to assess and select specific impact indicators on the innovation performance of biomedical industry (see Figure 1).

1. Government subsidy: due to the special characteristics of the biomedical industry, the cost and risk of carrying out innovative activities are obviously higher than those of other industries, the government invests in R&D subsidies not only to meet a certain amount of enterprise capital needs, help enterprises to reduce costs and avoid risks, but also combined with the enterprise’s financial flexibility to realize the effective allocation of resources, so as to safeguard the stability of the cash flow and to improve the competitiveness of the enterprise’s innovation and enterprise value (38). As the most direct way for the government to support the development of the biomedical industry, R&D subsidies are given to ensure their R&D funds, promote their R&D activities, and improve their innovation capability and corporate performance (12). However, government subsidy resources are limited, and the scale and continuity of subsidies will directly affect the innovation performance of biomedical companies.
2. Tax incentive: the biomedical industry, as a strategic emerging industry of hundreds of billions cultivated by China, has been steadily expanding in scale, significantly enhancing its innovation capability and improving its economic benefits, which plays an important role in driving economic development and promoting people’s livelihood and employment. Behind the booming development of the biomedical industry, the support of tax incentives plays an important role (8). As an important means of government fiscal policy, it can effectively increase enterprises’ investment in R&D in the long term and plays an important role in guiding and regulating the development direction of the biomedical industry. In recent years, in order to support and promote the R&D and innovation, China has increased the tax incentives for the industry (8). Tax incentives can, on the one hand, reduce corporate taxation, increase corporate cash flow, promote R&D investment and improve corporate profits; on the other hand, they can fully reflect the government’s concern and support intended for the related industries.
3. Financing method: Historically, major technological revolutions have been supported by financial capital. This is particularly true in the biomedical industry. For enterprises to carry out continuous R&D and innovation, they must rely on financing. The main methods of financing include equity-based and debt-based financing, both of which are low-cost and usually have a long-life span (9, 33). Which financing method should a biomedical enterprise choose? Usually, it is necessary to take into account the stage of development of the enterprise as well as its financial characteristics. In the founding period, the product is mainly in the laboratory stage, due to the high risk, so the intervention of funds often require a higher return, this time can be introduced into the venture capital (Venture Capital, VC); in the input period, the product is in the approval of the certification and industrialization of the pilot production stage, low revenue, long investment cycle, large demand for funds, and mainly long-term funds, this stage of the financing methods Including private equity financing (Private Equity, PE) etc.; in the rapid development period, the product is successfully listed, rapid growth in revenue, but the market development and subsequent research and development needs high cash input, so usually use bank borrowing, bond issuance, main board IPO or refinancing, etc.; in the maturity period, the product enters the mature state, the expansion of the enterprise scale make the incremental effect of subsequent projects diminishes and the demand for M&A expansion increases, thus M&A financing and equity cooperation can be considered (9, 33). According to the data of existing listed enterprises, the financing methods of Chinese listed biomedical enterprises are mainly debt-based financing and equity-based financing. Debt-based financing mainly includes long-term and short-term borrowing; equity-based financing mainly includes capital received from investors. Equity-based financing has gradually become an important financing method for biomedical enterprises.
4. Professional level: Specialized investment is the trend of financial institutions’ development. Specialized investment institutions have comprehensive and professional industry knowledge. As an emerging industry, the biomedical industry has a high degree of innovation, complexity and professionalism. Specialized financial institutions can screen out enterprises with investment value faster and better through their professional knowledge of relevant industries so as to reduce costs and investment risks. In addition, specialized financial institutions can form the brand effect of the industry through their own investment experience and high investment success rate, which can improve stakeholders’ trust in them, thus accelerating the link between financial institutions and stakeholders, improving efficiency and injecting more momentum into the development of emerging industries.
5. Institutional background: This topic focuses on two types of financial institutions with or without a government background. The objectives and investment strategies of financial institutions with governmental backgrounds are more different from those of financial institutions with non-governmental backgrounds. Financial institutions with a government background can give full play to the correct positioning of the government in industry selection, improve the implementation effect of government industrial planning, improve project screening ability, and enhance the development promotion of emerging industries. On the contrary, financial institutions with non-government backgrounds do not have the above-mentioned advantages in the process of selecting investment industries, targets and strategies.

## 3 Study design

### 3.1 Research methodology of QCA and NCA

In this study, a qualitative comparative analysis (QCA) method for detecting sufficient causality was first used to explore whether the antecedent factors (“political” and “financial” combinations) could adequately produce the outcome (innovation performance of biomedical enterprises). The research methods of QCA can be divided into clear set QCA (csQCA), multi-value set QCA (mvQCA) and fuzzy set QCA (fsQCA) (39). Considering that fuzzy set qualitative comparative analysis (fsQCA) has the advantage of dealing with partial affiliation and degree change problems compared with the other two categories, this study chooses the fuzzy set qualitative comparative analysis (fsQCA) method to explore the full causal mechanisms of the configuration effects of the “political” and “financial” elements on the innovation performance of biomedical enterprises. fsQCA adopts a holistic perspective and conducts cross-case comparative analysis to explore the causal complexity of which groups of condition elements cause the emergence of expected outcomes and which groups cause the lack or absence of expected outcomes (40). For the biomedical industry, different sets of industry policy and financial institution may have diverse and complex effects on the innovation performance. Second, fsQCA method not only makes up for the shortcomings of the qualitative research method by using a large sample set of cases to solve the problems of applicability and uniqueness of traditional qualitative analysis but also makes up for the shortcomings of the quantitative research method by using a large sample for individual phenomena. Finally, this paper focuses on the “configuration effect” between the elements of “policy” and “finance” and the “interaction” among different indicators to find the best way to improve innovation performance.

In order to more fully explain the causality of the variables in this study, in addition to the fsQCA method that detects sufficient causality mechanisms, the NCA method was used in this study to analyze the necessity of causality among the study variables (41, 42). NCA is a research methodology and data analysis methodology based on the logic that conditions may be necessary but not sufficient for an outcome to occur. The methodology triggers a new way of thinking about theory based on the logic of necessity, and thus research using NCA can provide interesting theoretical contributions. Second, because necessary conditions act independently of other causal structures, theoretical models of necessity can be simple. Typically, NCA researchers will use theoretical models with only one or a few potentially necessary antecedents to test for the necessity of antecedent variables to cause a research outcome. Finally, this approach complements other methods that are not based on necessity logic, such as regression analysis, QCA analysis, etc. QCA focuses primarily on sufficiency analysis, i.e., identifying multiple combinations of conditions that are sufficient to satisfy the outcome. Currently, it is often recommended that a necessity analysis for QCA precede a sufficiency analysis, but necessity analyses are often missing from specific applications of QCA in business and management. Compared with the fsQCA method, the NCA approach not only detects whether a condition is necessary for the outcome to arise but also shows the degree of necessity of this condition and can explain the importance of the condition variables more precisely and deeply (43, 44). Therefore, the combination of fsQCA and NCA can not only test the influence of specific “political” and “financial” groupings on innovation in biomedical enterprises but also reflect the important degree of specific “political” and “financial” factors.

### 3.2 Case selection

In 2010, the State Council issued the Decision on Accelerating the Cultivation and Development of Strategic Emerging Industries and publicly released information on listed companies since 2010, but the information is incomplete. Starting from 2012, the data related to corporate innovation and R&D of listed companies are complete. Therefore, this study selected A-share listed companies in Shanghai and Shenzhen in the biomedical industry from 2012 to 2020 as the research object. According to the SFC 2020 industry classification standards, as of July 24, 2021, there are 145 enterprises in the biomedical industry listed on A-shares in Shanghai and Shenzhen in China. This study uses these listed enterprises as the research sample and further screens: (1) exclude enterprises with special treatment such as ST and ST*; (2) exclude enterprises with industry policies, investment and financing and patent data from 2012 to 2019 incomplete enterprises, and finally determine the valid biomedical manufacturing listed enterprises as 60. The data obtained in this study are from annual reports of listed enterprises, the Wind database and the CSMAR database.

### 3.3 Variable definition

#### 3.3.1 Explanatory variables

1. Government subsidy: In terms of the scale of government subsidies, this study intends to choose government subsidy related to R&D and innovation of enterprises, including special fund support for independent innovation, technological reform support funds, government awards and patent application grants (4). The ratio of the total amount of government subsidy received by listed biomedical enterprises to their total operating revenue is used as an indicator to measure the scale of government subsidy (6).
2. Tax incentive: China’s tax incentive for enterprise technology innovation is mainly reflected in the corporate income tax section. According to the study of Liu, the ratio of the total amount of tax rebates received by listed biomedical enterprises to their total operating income is selected as a measure of the tax incentive received by enterprises (45).
3. Financing method: This study selects debt-based financing and equity-based financing for in-depth study. Debt-based financing mainly includes short-term borrowing and long-term borrowing; equity-based financing mainly refers to the funds obtained through the change of share capital, such as the issuance of additional shares. The measure of financing method is measured by the ratio of the total amount of equity-based financing to the total amount of bond financing. The smaller the value, the more the proportion of enterprises choosing the debt-based financing method, and the opposite, the more the proportion of equity-based financing.
4. Professional level: This study focuses on whether specialized and non-specialized financial institutions have a biomedical background and have invested in biomedical-type projects. The professional level of the top 10 shareholders in the annual report is selected as the indicator of this study, and the more shareholders with a professional background, the greater the indicator of professionalism.
5. Institutional background: This study focuses on the number of financial institutions with state-owned and government backgrounds among the top 10 shareholders in the annual report as a measure.

#### 3.3.2 Explained variables

Innovation performance: In studies related to the innovation performance of enterprises, since it is difficult to measure the quantity and quality of innovation output directly, most scholars use patent-related indicators to study it instead. As mentioned above, the biomedical industry is characterized by high risk, high investment, high technology and long cycle time, so the approval and granting of patents usually takes a long time and has a serious lag. For this reason, this study uses the number of patent applications with a high innovation rate to measure the innovation performance of enterprises, based on the research of related scholars (46). Specifically, considering the different years of establishment of listed biomedical enterprises, this study chooses to use the total number of invention patent applications of listed biomedical enterprises since 2012 to measure the innovation performance of the enterprises.

In summary, the variables that affect the innovation performance of biopharmaceutical companies in China under different “political” and “financial” grouping models are defined and measured as follows, first in Table 1.

**Table 1:** Composition of indicators and measurement methods of study variables.

Variable name	Symbols	Variable description
Government Subsidy	Gov	Total government subsidies/total operating revenues
Tax Incentive	Tax	The total amount of tax refunds/total operating revenues
Financing Method	Fin	The total amount of equity financing/Total amount of bond financing
Professional level	Pro	Number of institutions with a biopharmaceutical background and investment in biopharmaceutical projects
Institutional Background	Ins	Number of state-owned and government background investment institutions
Innovation Performance	Info	Total number of invention patent applications

### 3.4 Variable assignment and anchor point determination

First, to ensure the reliability and validity of the measurement of variables in this study, the measurements of variables were selected from established studies by existing scholars and reasonably modified according to the purpose of this study. Second, in order to accurately reflect the inter-case variability, with reference to previous studies, the three calibration points of the five independent variables with one respondent variable fully affiliated, crossover point, and fully unaffiliated were set as the upper and lower quartiles of descriptive statistics in this study, which were 75% fully affiliated, 50% crossover point, and 25% fully unaffiliated (39). Fiss’ study suggested that in the fsQCA anchor point determination and fuzzy value calibration, there is a possibility that the anchor points may have the same value as the original data during the process of fsQCA anchor point determination and fuzzy value calibration (40). Therefore, to avoid this situation, this study further reviewed the data and increased the calibration points where the same values occurred by 0.001 while ensuring that the maximum value did not exceed 1. The results of descriptive statistics and calibrated anchor points for each variable in this study are shown in Table 2.

**Table 2:** Descriptive statistics and variable calibration anchor points.

	Descriptive analysis				Fuzzy set calibration		
Variables	Average value	Standard deviation	Minimum value	Maximum value	Completely unaffiliated	Delivery almost	Fully affiliated
Gov	5.902	17.640	0.000	98.109	0.254	0.707	1.984
Tax	0.409	1.447	0.000	10.667	0.001	0.003	0.201
Fin	9.528	26.917	0.000	180.334	0.785	1.920	5.603
Pro	2.000	1.414	0.000	5.000	1.001	2.001	3.001
Ins	1.717	1.595	0.000	7.000	0.001	1.500	2.001
Info	49.783	76.644	1.000	397.000	6.001	14.000	56.250

## 4 Research results and analysis

### 4.1 Analysis of necessary conditions

The NCA method identifies whether the study variable is a necessary condition and detects the effect size of the necessity condition. The effect size is indicated by the bottleneck level in the NCA method. Dul’s study indicates that the bottleneck level value ranges from 0 to 1; when the value is less than 0.1, it means that the effect size is too small; on the contrary, when the value is closer to 1, it indicates that the necessity effect size is larger (41). Regression (CR) and ceiling envelopment (CE) can be used to deal with different levels of discrete variables as well as continuous variables. The CR method is chosen if the variables in the study are all discrete or continuous variables and are at or above level 5; the CE method is chosen if the variables in the study are dichotomous or do not reach level 5. The CR or CE method allows the corresponding functions of the variable relationships to be obtained and the effect sizes to be analyzed accordingly. According to Dul’s study, in the NCA method, two conditions are required to satisfy the necessary conditions, which are that the effect size (d) is greater than or equal to 0.1 and that the results of Monte Carlo simulations of permutation tests show significant (41).

In this study, the effect sizes of the variables were calculated using both CR and CE methods (see Table 3), and the results of the NCA test showed that in the “political” and “financial” dimensions, the results for tax incentive and financing method were significant, but the effect sizes were too small to be identified as a necessary condition to influence innovation performance (41). In addition, government subsidy (*p* = 1.0), professional level (*p* = 1.0), and institutional background (*p* = 1.0) are not significant, indicating that they are also not necessary for innovation performance. In addition, in the bottleneck analysis, the bottleneck level indicates the range of the maximum observed level values that the antecedent conditions need to satisfy when the level of the maximum observed range of the results is met, and the specific results of the bottleneck analysis in this study are shown in Table 4. the results of the data show that if the 60% level of innovation performance is to be achieved, the 0.8% level of tax incentive and the 0.3% level of financing method are needed, and the other three dimensions of “government” and “finance” do not have bottleneck levels.

**Table 3:** Analysis of results of necessary conditions of NCA method.

Conditional variable	Methods	Accuracy	Upper limit area (Ceiling zone)	Scope	Effect size(d)^b^	*p* value
Gov	CR	100%	0.000	0.098	0.000	1.000
	CE	100%	0.000	0.098	0.000	1.000
Tax	CR	100%	0.014	0.096	0.014	0.069
	CE	100%	0.018	0.096	0.018	0.061
Fin	CR	100%	0.003	1	0.004	0.098
	CE	100%	0.007	1	0.008	0.094
Pro	CR	100%	0.000	1	0.000	1.000
	CE	100%	0.000	1	0.000	1.000
Ins	CR	100%	0.000	0.099	0.000	1.000
	CE	100%	0.000	0.099	0.000	1.000

^a^Calibrated fuzzy set affiliation values. ^b^0.0 ≤ d < 0.1: “low level”; 0.1 ≤ d < 0.3: “medium level.” ^c^The permutation test (permutation test, re-sampling) in NCA analysis. (number of times = 10,000).

**Table 4:** NCA method bottleneck level (%) analysis result.

Info	Gov	Tax	Fin	Spe	Ins
0	NN	NN	NN	NN	NN
10	NN	NN	NN	NN	NN
20	NN	0.2	NN	NN	NN
30	NN	0.3	NN	NN	NN
40	NN	0.5	NN	NN	NN
50	NN	0.6	0.1	NN	NN
60	NN	0.8	0.3	NN	NN
70	NN	1.0	0.5	NN	NN
80	NN	1.2	0.6	NN	NN
90	NN	1.3	0.7	NN	NN
100	NN	1.5	0.9	NN	NN

^a^CR method, NN, unnecessary.

In fsQCA, a “necessary condition” means that the condition always occurs when the result is present, and if it does not occur, the result cannot be generated. In general, an antecedent condition is considered necessary for the outcome variable when the consistency is greater than 0.9 or close to 0.9 (41). The consistency of all the antecedent conditions in this study is less than 0.9, which indicates that none of the antecedent variables in this paper is necessary to satisfy the high/low to medium innovation performance (see Table 5). This also indicates that the effects of industry policy and financial institutions on innovation performance of biomedical enterprises are more complex and are the result of a combination of variables that cannot be explained by a single variable independently, and further analysis of the variables, i.e., group analysis, is required.

**Table 5:** Results of the necessity test for the condition variable of the fsQCA method.

Conditional variables	Result variables	
	High innovation performance (Ino)	Low to medium innovation performance (~Ino)
Gov	0.64	0.42
~Gov	0.47	0.68
Tax	0.50	0.41
~Tax	0.55	0.64
Fin	0.49	0.55
~Fin	0.60	0.54
Pro	0.57	0.38
~Pro	0.49	0.67
Ins	0.68	0.53
~Ins	0.40	0.55

### 4.2 Configuration analysis

After calibrating each element, the truth table was further constructed to obtain different configurations of cause conditions. Douglas pointed out that in small sample studies, researchers can consider a minimum case frequency of 1 or 2 (41). Therefore, in this study, fsQCA 3.0 software was used to analyze the case data of 60 listed biomedical enterprises, and the case frequency of no less than 1, consistency greater than 0.8, and PRI Consistency greater than 0.75 as the judgment criteria to obtain the group paths that produce high and low to medium innovation performance results, and to name each group. Specifically, the analysis results of QCA show that there are four groups that produce high innovation performance, namely S1, S2, S3 and S4, and the consistency index of all four groups is greater than 0.85, which has high consistency and is sufficient condition for high innovation performance; there are two groups that produce low to medium innovation performance, namely NS1 and NS2, and the overall consistency index is 0.86, which has high consistency (see Table 6). Each of the grouping paths affecting the innovation performance of biomedical enterprises will be analyzed in detail below.

**Table 6:** High/non-high innovation performance grouping of biopharmaceutical companies.

	High innovation performance				Non-high innovation performance	
Variables	S1	S2	S3	S4	NS1	NS2
Gov		●		●	⊗	⊗
Tax	●	●	●	●	⊗	⊗
Fin	⊗	●	⊗			
Pro	●	●			⊗	●
Ins	●	⊗	⊗	●	●	⊗
Consistency	0.95	0.97	0.86	0.91	0.87	0.93
Original coverage	0.21	0.05	0.21	0.15	0.18	0.13
Unique coverage	0.18	0.03	0.08	0.06	0.07	0.03
Overall consistency	0.95				0.86	
Overall coverage	0.23				0.43	

●, core condition present. ⊗, core condition is missing; ●, marginal condition is present. ⊗, Marginal condition is missing; “blank” means that the presence or absence of the condition does not affect the result.

#### 4.2.1 The “policy” and “finance” synergistic configuration that generates high innovation performance of biomedical enterprises

S1 is driven by the synergy of tax incentive, professional level and government background. S1 shows that the synergistic path with high tax incentive, high professional level and high government background of financial institutions and non-high equity-based financing methods as the core conditions can produce high innovation performance. The S1 group indicates that in the case of biomedical enterprises with a low percentage of equity-based financing, they need to actively understand the government tax incentive and choose a financial institution with a government background and high professionalism. There may be the following reasons for this grouping path: the introduction of financial institutions with strong professionalism and high government background significantly enhances the professionalism and related government resources for biomedical enterprises, which provides sufficient preparation and platform for the subsequent innovation development of enterprises and has a strong role in promoting the innovation performance of enterprises. On the other hand, in order to avoid excessive involvement of financial institutions or even interference in the development of enterprises, biomedical enterprises choose lower equity-based financing as a protection of their own voice and control rights. Therefore, balancing financial institutions and enterprises’ own shares while grasping policies and enjoying tax benefits becomes an important way and direction for biomedical enterprises’ innovative development.

S2 is driven by the synergy of government subsidy, financing method and the professional level of financial institutions. S2 shows that the synergistic combination of high government subsidy, high equity-based financing, high professionalism of financial institutions, and non-high government background as the core condition complementing high tax incentive as the marginal condition can produce high innovation performance of biomedical enterprises. The S2 grouping path indicates that biomedical enterprises can achieve high innovation performance if they bring in professional and non-government financial institutions for equity-based financing while fully enjoying the policy incentive. This path is consistent with the S1, both of which reflect the important influence of government policy support on biomedical innovation performance. The difference is that while enjoying government policy support, introducing professional financial institutions and high equity-based financing, biomedical enterprises need to focus on choosing non-government background financial institutions for financing, which may be due to the fact that higher equity-based financing dilutes the equity of the enterprise’s original shareholders and reduces the control. Therefore, in order to ensure that enterprises enjoy government support and a large percentage of equity-based financing while retaining self-ownership, financial institutions with non-high government backgrounds are a better choice.

S3 Tax incentive and debt-based financing are synergistically driven. S3 shows that a synergistic configuration of high tax incentive and debt-based financing as the core condition complementing the marginal condition of non-government background financial institutions can produce high innovation performance. This histogram once again reflects the importance of tax incentive to the innovation development of. The reason for this path may be that with a high percentage of government tax incentive, the enterprises’ demand for capital is alleviated, and therefore, they prefer debt-based financing, and debt-based financing from non-government financial institutions is relatively better than government financial institutions in terms of application process and flexibility.

S4 tax incentive and government background driven. Configuration S4 shows that a synergistic “government” and “financial” configuration with high tax incentives and high government background financial institutions complementing high government subsidy as marginal conditions can produce high innovation performance of. This configuration once again demonstrates the importance of policy support. In addition, with government subsidy and tax incentive, biomedical enterprises can enhance their innovation performance by choosing financial institutions with high government backgrounds.

#### 4.2.2 The “government” and “finance” synergistic grouping that generates low to medium innovation performance of biomedical enterprises

Through the analysis of the data, this study also detects two paths of the “policy” and “finance” histories that generate low to medium innovation performance in biomedical enterprises. First, the results of the NS1 pathway suggest that in the absence of high tax incentive, lack of high professional level of investment institutions, and insufficient government subsidy in collaboration, the innovation performance of is not high even if the participating institutions have a high government background. In addition, group NS2 shows that in the absence of high tax incentive, lack of high government background of investment institutions and insufficient access to government subsidy, the innovation performance will not be high even if the investment institutions have a high level of professionalism. For the two grouping paths that fail to generate high innovation performance, this study finds that government policy support plays a very important role in the innovation performance of biomedical industry, even if the financial institution has a high degree of professional and government background, as long as the biomedical enterprises do not enjoy sufficient government subsidy and tax incentive, it will lead to a situation of low innovation performance of. It can be seen that government support for emerging industries through policies becomes an important backing force for innovation development.

### 4.3 Robustness tests

Checking the robustness of the analysis results is a key step in a QCA study. In this study, the data were analyzed again after adjusting the case frequency to 2 and the consistency threshold to 0.81 to compare the changes in the groupings to assess the results. After testing, it was found that the combination of pathways affecting the innovation performance of biomedical enterprises did not lead to substantial changes in the number, components, consistency and coverage of the histories after the parameter adjustment. Therefore, it was concluded that the analytical results obtained in this study were reliable and robust.

## 5 Research conclusion and outlook

### 5.1 Research findings

The stability and competitiveness of the industry collaboration is the core of China’s biomedical industry development. As mentioned above, China has made the biomedical industry as the first national strategic emerging industry, in order to accelerate the construction of biomedical power, in recent years, the state and the region frequently released a series of reform policies, and actively promote the leapfrog upgrading of the biomedical industry, which is particularly important for linkage and collaboration between the pharmaceutical R&D centers, manufacturing enterprises, hospitals, the government, investment entities and other subjects are particularly important. This paper analyzes the relationship among industry policy, financial institution and innovation performance in the biomedical industry from the configuration perspective, combining necessary condition analysis (NCA) and qualitative comparative analysis (QCA) research methods, using the A-share listed enterprises in Shanghai and Shenzhen in the biomedical industry from 2012 to 2020 as the research objects. The research findings indicated that: (1) the results of the NCA study show that neither individual industry policy nor the characteristics of financial institutions constitute a necessary condition for high innovation performance of biomedical industry, but increasing government tax incentive and increasing the proportion of equity-based financing play a more significant role in improving innovation performance in China. Globally, the health industry’s status as a sunrise industry is based on the technological possibilities offered by the continuous development of biotechnology, the large consumer base provided by an aging society, and the large sums of money paid for by increased government welfare spending and policy support, which constitute the favorable factors for the development of the health industry (47, 48). Among them, the rapid development of science and technology has become a key force in the development of the health industry worldwide. Breakthroughs and research in biological and cellular biochemical science and technology have greatly reduced the cost of health products and services, and enhanced the industry’s competitiveness and affordability. In addition, in the world’s top 500 multinational biomedical enterprises, R&D investment accounted for 10 to 15% of its sales revenue. In United States, life and health industry added value of about 18% of the proportion of GDP, of which health services accounted for 65% and the growth rate of 70%; in the European Union, Japan, Canada, the life and health industry added value of more than 10% of the proportion of GDP, of which the city of Kobe, Japan, has become a world-renowned city of medical industry. Even so, due to the specificity of the biomedical industry, the realization and further improvement of the performance of innovation cannot be achieved without collaboration of “industry-academia-research-government-finance,” leave any of these, the development goal of the biomedical industry will not be achieved (47, 49). (2) The results of the QCA study show that there are four grouping paths that can generate high innovation performance, and each of these four groupings presents multiple combinations of ways to achieve high innovation performance. This result indicates that in China, biomedical enterprises can compare the four grouping paths to achieve high innovation performance according to their own characteristics and choose the path that best fits their future development in terms of industry policy and financial institutions to achieve high innovation performance. Finally, tax incentive is included in all four high innovation performance groupings, and government subsidy and tax incentive are included in two of the low to medium innovation performance groupings, indicating that government guidance, support, and assistance play a very important role in the development of biomedical industry in China. Similar to the results of this study, in order to stimuli the innovation performance, several large global countries promote the innovation and development of the biomedical industry by means of policy support (47). For example, Russian science and technology forecasts do focus sufficiently to promising technologies in biomedical industry, nanotechnology, and medical technology (11). The United States federal government provides large amounts of research funds for R&D in the biomedical industry. As early as the 1970s, the U.S. federal government’s R&D investment in the biomedical field already accounted for 11% of its total R&D investment. Since the 20th century, the U.S. federal government has been spending about half of its total non-defense R&D on health and human services (50). According to the latest White House budget, the federal government will spend about $38.5 billion, $40.8 billion, and $37.9 billion on health and human services R&D in 2019, 2020, and 2021, respectively, with a large portion of the funding going to support R&D in the biomedical industry (50). At present, the European Union does not have specific funding for biomedical technology innovation, but rather includes it in a broad spectrum of support programs for scientific research, such as the Marie Curie Fund. Since 1991, the EU has supported a total of 2,629 projects, of which 116 were in Industrial Technologies and 75 in Fundamental Research. In addition, the EU has initiated a number of studies on the biotechnology innovation environment and innovation policies in EU member states (18).

### 5.2 Research contributions

The stability and competitiveness of the biomedical industry chain is the core of the development of China (3). As mentioned above, China has taken the biomedical industry as the first national strategic emerging industry. In order to accelerate the construction of biomedical power, in recent years, the state and the region have frequently issued a series of reform policies to actively promote the biomedical industry leapfrog upgrading, in which the linkage and collaboration among pharmaceutical research and development centers, manufacturing enterprises, hospitals, governments, investment entities and other subjects are particularly important (8). Existing studies also point out that the development of national strategic emerging industries cannot be separated from the support and influence of the external environment system, especially the joint influence of “industry,” “academia,” “research,” “government,” and “finance” (9). Most of the studies focus on the single dimension to explore the impact on enterprise’s innovation (4). However, measuring the external environment that affects enterprise’s innovation requires a holistic, group perspective and a more comprehensive research approach. Therefore, this study analyzes the impact of the configuration effect between the dimensions of “policy” and “finance” on the innovation performance of biomedical industry. The results of this study aim to provide theoretical and practical contributions to the research on innovation development of national strategic emerging industries.

#### 5.2.1 Theoretical contributions

Firstly, this study, for the first time, includes the industry policy and financial institution aspects into the same theoretical model to explore the configuration effect of these two subjects on the innovation performance of biomedical industry.

Secondly, this study selects A-share listed enterprises in Shanghai and Shenzhen in the biomedical industry from 2012 to 2020 as the research subjects and adopts the NCA method to test the causal relationship between the necessity of a single dimension of “government” and “finance” to generate high innovation performance, which is representative. The results found that no single dimension could meet the necessity criterion, suggesting that individual dimensions do not constitute a bottleneck for high innovation performance. Although a large number of existing studies have demonstrated that individual policy preferences or financing dimensions are significantly associated with innovation performance in emerging industries, this study finds that these dimensions are not necessary conditions for generating high innovation performance. Therefore, it is important for policy-making and financial institutions affecting the biomedical industry to develop synergies and find suitable grouping paths to improve the innovation performance.

Finally, this study uses a combination of QCA and NCA to analyze the necessity and adequacy of the “political” and “financial” grouping to generate high innovation performance. In recent years, in the field of sociological research, the combination of QCA and NCA approaches has been widely used to explore the possibility of the occurrence of causality in the group state, but the relationship between the external environment, especially industrial policies, financial institutions and enterprise’s innovation performance, has not yet been studied. In particular, the QCA method is suitable for analyzing the complex causality of sufficient conditions, which is very suitable for analyzing the relationship between the “political” and “financial” groups and innovation performance in this study, while the NCA method can analyze the causality of necessary conditions in a more detailed and clear way. The NCA method can analyze the necessary causality in a more detailed and explicit way, so it is very suitable for analyzing the individual correspondence between the “political” and “financial” dimensions and innovation performance. This study is the first to combine the two approaches and apply them to the biomedical field, which can help promote the development of the relationship between the external environment of “policy” and “finance” and the innovation performance of emerging industries.

#### 5.2.2 Practical contributions

This study explores and verifies the relationship between the configuration effect of “policy” and “finance” and the innovation performance of biomedical enterprises based on the perspective of industry policy and financial institutions, which provides a new perspective for the development environment of the biomedical industry in China and inspires. The practical contributions of this study include: (1) the development of China’s strategic emerging industries cannot be separated from policy guidance, support and increasing government subsidy, and tax incentive are an important measure to promote the innovative development. S3 and S4 both show that when the input strength and professional dimension of financial institutions are not good, the government can effectively promote the innovation performance through policy subsidy, tax incentive and investment institutions with government background. (2) In S1, S3 and S4, tax incentive is all core condition and also appear as marginal conditions in S2. This shows that tax incentive in the biomedical industry significantly promote the innovation performance of enterprises, and they play a more prominent role as one of the methods of government policy support compared with government subsidy. (3) The two paths S1 and S2 show that the financing method and financial institution background present mutually exclusive grouping results, which shows that biomedical enterprises prefer financial institutions without government background when financing in the form of equity-based method; on the contrary, they prefer financial institutions with government background when choosing the financing method mainly in the form of debts. The mutually exclusive results of these two dimensions once again reflect that value autonomous control and that with high flexibility can better promote innovation with sufficient capital. (4) S3 is the simplest combination of the four grouping paths, and this path shows that biomedical industry can achieve the goal of high innovation performance by choosing debt-based financing from non-government background financial institutions while taking full advantage of government tax incentive. This phenomenon reflects the side that, with sufficient funds, the biomedical industry has paid much attention to the importance of innovation development and all working toward it.

### 5.3 Research gaps and future research

There are three main shortcomings of this study, which can be further improved and expanded in future studies. First, due to the limitation of data availability, only 60 listed biomedical enterprises were selected as the sample of this study, which affects the accuracy and generalizability of the findings to a certain extent. Future studies can target more enterprises, different emerging industries and data related to innovation, and conduct more in-depth research on how to improve innovation performance. Secondly, this study adopts a qualitative comparative analysis method and tries to explore the impact of the configuration effect of “policy” and “finance” on enterprise innovation performance from the perspective of grouping, but it is still challenging to deepen the grouping path and conduct qualitative research. In addition, whether the study of multiple cases is representative of large sample data is also an important issue to be considered. Moreover, the possibility of quantitatively measuring the influence of government policies and financing institutions is important. It would also be beneficial to incorporate references to works on other countries where an effort has been made to measure this influence. Therefore, future studies can try to adopt multiple research methods to further verify and improve the accuracy of the results. Finally, in addition to the configuration effect of “policy” and “finance” on the innovation performance, “industry,” “academia,” and “research” can also have an impact on the innovation performance. The path to finding the antecedent variables and the path to the best innovation performance is an important direction to be studied in the future.

## Data availability statement

The raw data supporting the conclusions of this article will be made available by the authors, without undue reservation.

## Author contributions

YZ: Writing – original draft, Writing – review & editing.
